# “Mind the Gap” - An overview of the role of the Extensions Community Healthcare Outcomes (ECHO) model in enhancing value in health care delivery

**DOI:** 10.3934/publichealth.2023008

**Published:** 2023-02-22

**Authors:** Christina Kenny, Anushree Priyadarshini

**Affiliations:** Faculty of Business, Technological University Dublin, Aungier Street, Dublin 2, Ireland

**Keywords:** ECHO, patient reported outcomes measures, healthcare management, telemedicine, e-health, digital healthcare

## Abstract

The ECHO (Extensions Community Healthcare Outcomes) model of healthcare delivery has grown rapidly since its establishment and increased in popularity in recent years. This expansion has developed alongside the growing incidence of chronic diseases and the need to better manage them. The increasing uptake in ECHO has presented a requirement to assess its true value as healthcare costs are increasing globally, resulting in a growing demand by governments and policy makers to ensure chronic disease management strategies provide true value. Therefore, the aim of this review is to examine the impact that ECHO has on clinical practice and how such impacts are measured or evaluated. A narrative literature review is carried out to examine the outcomes assessed in ECHO-related studies. Three key academic databases were utilised for the literature search: Web of Science, PubMed, and Medline. Keywords relating to the review were chosen and searched for. Papers were screened using specified inclusion and exclusion criteria relating to years of publication (2000–2020), type of publication (original research, review papers and meta-analyses) and language requirements (English language only). This review found that while the ECHO model is expanding, and improving the so-called “knowledge gap” between specialists and primary care physicians, there is also a gap in the ways value is examined within ECHO. Most studies on ECHO lack an examination of patient reported health outcomes and appropriate, comparative costing methods. Current ECHO-related studies lack vital components that demonstrate the value of the model. Such components include patient reported health outcomes and detailed costing comparisons between the ECHO model and the traditional care pathway it is replacing.

## 1. Introduction

Chronic disease incidence rates are at unprecedented levels. In 2020 chronic disease contributed to some 73% of global deaths [1]. The increasing complexities associated with chronic conditions require more specialised, multidisciplinary medical teams and a more integrated approach to care [2]. Alongside the growing burden of chronic disease, multimorbidity is becoming an increasing issue as life-expectancy also continues to increase. Multimorbidity is defined as being “the coexistence of two or more chronic conditions” [3]. Most people living with one chronic condition, will often have associated co-morbidities [4]. As non-communicable diseases reach epidemic proportions [5] so too does the associated cost and the pressure they put on healthcare infrastructure worldwide. Both spending on chronic conditions and their overall costs are increasing [6]. Multiple morbid conditions often result in patients availing of more hospital resources, pharmacological interventions and even admissions [7]. Rapoport et al. [8] found that appointments with doctors increase by a half per each chronic condition experienced by a patient.

The World Economic Forum [9] predicts that chronic disease may cost as much as USD 47 trillion by 2030 and as such there is an increasing need to better manage chronic disease. While most people in the developed world have access to a general practitioner in a primary care setting, specialty care is not as widely accessible [10]. It has been well-discussed and documented in the literature that rural areas lack the same healthcare resources that their urban counterparts [11]. Medical diversity is another issue faced in rural medicine. In 2004, 41% of physicians in rural areas of 10,000 people or less were family physicians [12]. These factors have acted as motivating drivers for the formation of the ECHO (Extension in community healthcare outcomes) project and hence the ECHO model was born out of these necessities in the early 2000s.

The concept of the ECHO model is a simple one. The model centres around an expert or a team of experts such as a consultant or multidisciplinary team. This expert or team of experts conduct regular telehealth meetings on an online video platform which non-expert physicians can join to learn more about a particular disease, ask questions or even present patient case-studies for expert input [13].

**Figure 1.**
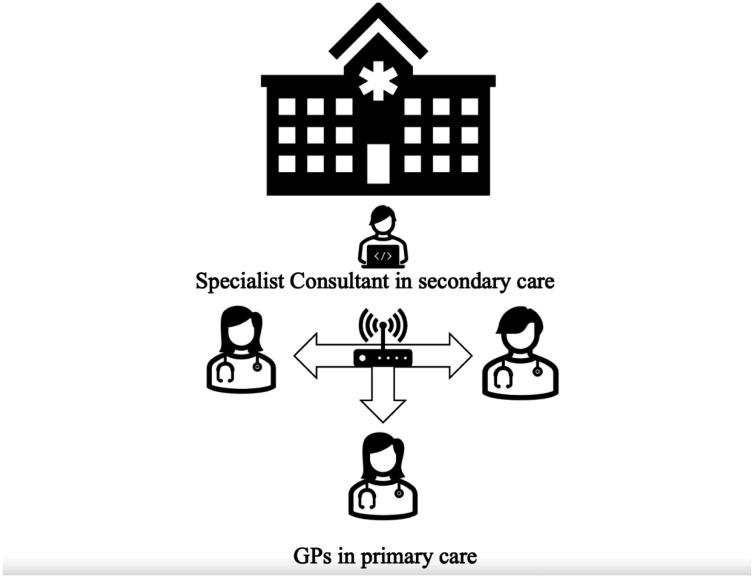
A configuration of the ECHO model (Concept illustration by authors).

Due to the challenges faced by rural medicine, the ECHO model was initially established by Dr Arora to better manage the growing prominence of the Hepatitis C Virus (HCV) in New Mexico amongst underserved and rural communities. New Mexico is a largely rural state [13] and as such, patients in remote areas often did not have access to specialist healthcare. The HCV epidemic originated from mass infection in the 1970s and 1980s [14]. By the early 2000s numerous people were left without specialist care due to their rural location. Before the commencement of ECHO, approximately 1,600 patients received care for HCV despite around 34,000 patients suffering with the virus [15]. The ECHO project began with specialist care teams holding weekly tele-ECHO meetings in which the specialist and community providers use video technology to communicate via a hub and spoke platform (Figure 1). Physicians can join from remote locations to learn from both each other and the specialists present. The tele-meeting was the first of its kind to provide an opportunity for rural physicians to troubleshoot and present complex medical cases. It enabled primary care physicians to treat patients they would have otherwise referred elsewhere [16]. The concept enabled thousands to receive specialist care from their own physician. The aim of ECHO was to deliver specialist care to those remote, underserved geographical areas that would otherwise be overlooked.

ECHO has since grown [17] and now spans over 4600 different programs and over 191 countries [18].

**Figure 2.**
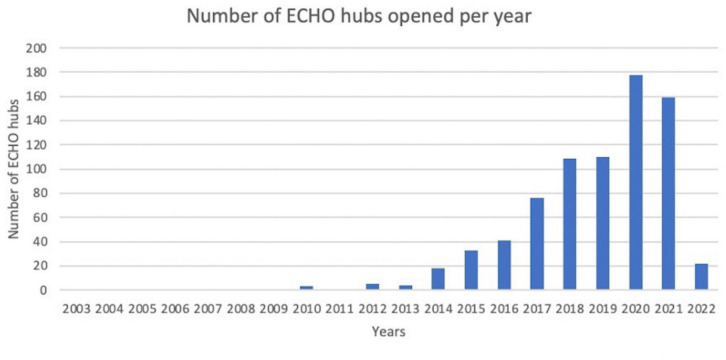
ECHO Hub launches in the US by year [18].

This growth warrants an in-depth analysis of the true impact of ECHO. This growth warrants an in-depth analysis of the true impact of ECHO. Therefore, the overall aim of this review is to examine common clinical outputs such as: healthcare outcomes, healthcare professional competencies and cost. In order to properly assess the true value provided by ECHO healthcare outcomes and compare it to other healthcare interventions, the cost of the intervention must be analysed.

## 2. Methodology

**Figure 3.**
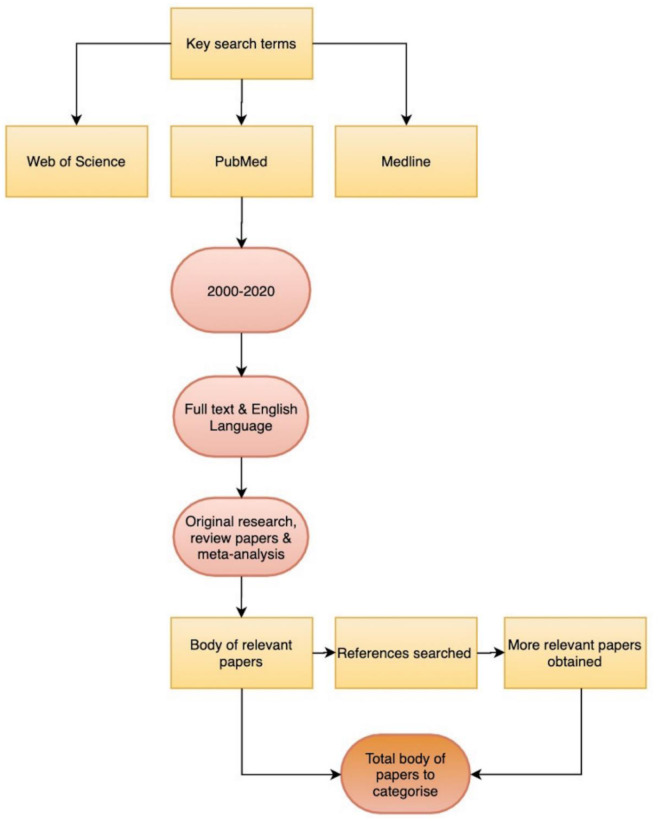
Search strategy (2000–2020) used within this review.

Papers for the review were searched on a range of reputable, academic databases (Figure 3) including Web of Science, PubMed and Medline using the following search terms: “ECHO model”, “Project ECHO”, “Extensions in Community Healthcare Outcomes”, “Extensions in Community Healthcare Outcomes project”, “telehealth” and “telemedicine”. Suitable papers were those relevant to the topic that met the inclusion criteria. Following the initial search, papers were hand-selected on reading the abstracts of papers. Inclusion criteria for this paper included: publication date between 2000–2020, full-text papers available in English and categorised as any of the following: original research, review papers and meta-analyses. If a paper met all of the inclusion criteria it was read in full by the author. Similarly, if there was any ambiguity surrounding a paper's suitability, it was also read in full. If the full text revealed that not all the inclusion requirements were present, the paper was excluded. Suitable papers were read in full and then categorised into their relevance to the review. Additional literature was then acquired using the snowball method in which already-included literature led to more relevant papers.

A framework with two broad categories was predefined: clinical disease applications and the impact of ECHO, which included the previously defined outcomes of the model - the clinical outcomes of the patient, the impact ECHO has on professional competency and ECHO's potential for cost-saving. An individual paper could be categorised into more than one field. The review was further expounded by reading each category separately and regrouping papers where appropriate. Data obtained for certain statistical information were gathered by directly targeting appropriate websites [1],[5].

## 3. Results

ECHO has been used in a wide range of chronic diseases across numerous clinical areas. Some of its applications are:

### 3.1. A tool in chronic disease management

Using Hepatitis C as a model [15] ECHO branched into other disease areas, including chronic disease. Due to the complex, wide-ranging and highly specialised nature of chronic disease, ECHO provides clinicians in primary care with the expertise and education to treat patients without referral elsewhere.

ECHO has been widely utilised in the treatment of chronic pain. Chronic pain is defined as pain that lasts longer than six months [19]. Furlan et al [20] evaluated the effect ECHO had on primary physician's ability to manage chronic pain in patients within their rural areas. The ECHO hub was operated by a multidisciplinary team of physicians from various clinical backgrounds including pain medicine, family practice, neurology, psychiatry, and addiction. This panel of experts is typically the same across the discipline of chronic pain. The weekly clinic was two hours long in which an expert presented or participating rural healthcare professionals could present their own case-study. The outcome measures for this study included self-efficacy and utilised pre and post comparisons. The study demonstrated a significant improvement in both knowledge and self-efficacy.

These differences in outcomes in the utilisation of ECHO for chronic pain might be attributed to a number of factors. Firstly, the length of the intervention or ECHO training might affect how successful it is in improving professional competency and efficacy. Some studies here have spanned several years while others have been less than a year. Similarly, as methods to improve the management of chronic pain globally have been increased [21] it is hard to suggest that ECHO is solely responsible for the improvement in management capabilities, but it has aimed to improve the chronic pain epidemic alongside other management strategies and policies [22].

Most of the ECHO programs targeting chronic disease are made up of a diverse multi-disciplinary team. As most cases of chronic disease are complex and comorbid in nature, an expert in the disease alone would not be sufficient to provide the necessary expertise to ensure thorough expertise on all that the disease presents [13]. There are now a wide range of ECHO programs targeting other chronic diseases not discussed in-depth in this review, these programs span the globe. As ECHO is being so widely employed within chronic disease management an in-depth understanding of its true impact in terms of delivering value is more important than ever.

### 3.2. Professional knowledge, efficacy, and the dissemination of information

The original aim of the ECHO model was to remove the barriers to specialist care for patients by educating and empowering primary care clinicians [13]. The removal of these barriers allows for knowledge dissemination, to educate primary care physicians, thus improving their overall self-confidence in their practice and thus, their efficacy. Almost every study examining the utility of ECHO examines some form of efficacy, knowledge, or confidence. While almost all studies employ these outcome measures, the methods used to examine them can differ widely. Self-efficacy tests are very commonly employed [23]. These surveys are often carried out pre and post-test for comparison. In other cases, a more objective form of pre- and post-assessment was carried out [24]. Self-reported confidence is also explored using surveys [25]. Not all tests are “self-reported” with some studies opting for more objective measures such as the KnowPain-12 scale. This survey is an adaptation of the knowPain-50 in which the test correlates with clinical behaviours and distinguishes between physicians with different levels of pain management expertise [26]. Some methods of outcome measurement are more objective than others. It is widely known that self-reported data is subject to bias [27] and as such, between the self-reported data and non-self-reported data, there are varying degrees of reliability, validity, and objectivity. While some act as an objective measurement of knowledge gained from the ECHO intervention [28], others are more self-reliant and subjective [20]. Although self-efficacy demonstrates improved self-confidence and self-belief in healthcare professionals, it has been demonstrated that physicians have a limited ability to self-evaluate [29] and as such, while this is an improvement, it may not necessarily translate into clinical relevance.

### 3.3. Patient clinical outcomes

ECHO allows for the breaking of barriers between primary and specialist care, the dissemination of information allows clinicians to become more educated in previously inaccessible expert care. As a result, patients too have improved access to previously untapped specialist care. This diffusion of knowledge and communication allows the closing of the treatment gap in many healthcare settings.

In general, studies have demonstrated that healthcare professionals engaging in the ECHO project feel more efficacious and confident and thus, this should translate into patient outcomes [30],[31]. However, very few studies have examined patient outcomes [32]. Most discuss how improved education should, in theory, translate to better outcomes but few directly demonstrate this.

Some studies do exhibit how they directly affect patients. When Katzman et al. [33] used ECHO as an educational tool for opioid prescribing, the study looked at specific metrics relating to the numbers of patients being prescribed opioid analgesics and co-prescribing of opioids per patient per year when compared to a comparison group. The number of patients benefitting from this can be seen by the sheer volume of patient cases being presented at each ECHO meeting. This demonstrated a clear, direct effect on patients. Similarly, some other studies have demonstrated the effect ECHO has had on mortality rates within certain complex, chronic disease. Viral response of patients with HCV has been compared in patients being treated by specialists and a group being treated by project ECHO healthcare professionals, both groups had similar outcomes [15].

### 3.4. A potential for cost-saving

ECHO could deliver expertise in a short space of time, quickly educating healthcare professionals on how to better manage and treat their patients. By being able to access care faster, it can eliminate the need for costly and time-consuming referrals elsewhere. While numerous studies imply that there are cost-savings, few analyse the cost-effectiveness of the ECHO intervention [34]. Many interventions don't directly discuss cot-effectiveness or cost-saving but do utilise other metrics that would imply downstream cost-savings. While in theory, ECHO should demonstrate a cost-saving, in some cases, the value of ECHO can be seen in other valuable ways. Generally, most studies that do explore the cost-saving potential of ECHO either demonstrate a clear saving or at least a saving in another, non-economical way [20],[31]. While this is typically the case, one study by Rattay et al [34] revealed that when the ECHO intervention was implemented in chronic HCV, it was more expensive than traditional care. The study discusses that this could be due to ECHO allowing for more targeted screening, higher adherence or improved access to treatment but were unable to confirm that this was the case.

### 3.5. The challenges of the ECHO model

While the ECHO model has allowed for the expansion of specialist care to otherwise deprived rural areas, it is not without its own challenges and limitations. ECHO faces barriers to its implementation which make uptake of the model limited to healthcare settings that can overcome these barriers. ECHO requires high-speed internet in order to operate. While this is not an issue for most of the developed world, it remains a problem for healthcare providers working in deeply rural communities around the world [35]. Similarly, ECHO faces logistical challenges. As ECHO relies on the input from a large multidisciplinary team of experts there may, at times be confusion over which care provider oversees certain patients [36] leading to disjointed patient care. Another potential challenge facing the future of the ECHO model is the onset of artificial intelligence in the management of chronic disease. Healthcare is moving towards more input from artificial intelligence sources which are becoming more readily available all over the globe making care more patient-focused and less centered around hospital care [37]. If the ECHO model does not integrate these changes into its future model, then it may fast become an outdated approach to chronic care management.

## 4. Discussion

This review examined the current applications of the ECHO model and how those applications might change in the future. It is clear from this review that ECHO, since it was first established has grown considerably in terms of clinical disease areas and also the impact it actually has on each of those clinical areas. As ECHO continues to expand and grow into more geographical areas and more clinical disease areas, it can be expected that more longitudinal studies will be available. As digital healthcare undergoes constant growth and investment as well as improved regulatory structures, all of which have been fast-tracked by the COVID-19 pandemic it can be expected that it too, will encourage the growth and dissemination of the ECHO model.

ECHO is working on closing the knowledge gap between rural and specialist care teams. The studies included in this review generally demonstrate improved clinician knowledge in terms of understanding symptoms, diagnosis and treatment within a particular disease area which leads to improved confidence and more efficacious work. However, it should also be noted that studies employ different metrics and means of measuring these outcomes and some are more objective than others, therefore comparing them might be inappropriate.

It is implied that by improving the standard of care provided to patients that, they too, will benefit from this. However, most studies do not actually explore the direct effect ECHO has on patients, their potential benefits are merely implied. This, therefore, cannot be used as definitive evidence that ECHO truly benefits patients in terms of improved clinical outcomes. One study [32] examined the impact ECHO had on patient outcomes and found it to be effective but needed more data to determine how efficacious it is. This study only included six papers that discussed patient outcomes and as such the data was limited. While ECHO is improving clinician's self-assessed outcomes, there isn't enough data to examine how patients are directly impacted, the inclusion of how ECHO impacts the health outcomes for any given disease within the standard sets prescribed by the International Consortium for Health Outcomes Measurement (ICHOM) should also be examined. More patient-centric studies are needed in order to obtain a holistic view of ECHO's value. Further clinical outcome measures should be employed including disease severity, impact on quality of life and impact on quality of life adjust years (QALY). Similarly, longitudinal qualitative studies are needed in order to assess the effects of ECHO, years after its first intervention in order to establish if the effects and adherence to its use is short-lived or sustainable long-term.

Most of the studies included here don't contain any form of cost-analysis or cost comparison between ECHO and the previous care pathway. Without a clear cost-analysis of any kind, it is difficult to calculate or examine ECHO's true value. It has been demonstrated that ECHO can be expensive to implement [34] but the exact reasons for this are unclear. Henceforth, future studies should include some analysis of cost-effectiveness.

## 5. Strengths and limitations of this review

This review provided a summary of current ECHO interventions being applied within chronic disease management as well their advantages and disadvantages. It also helped identify current gaps that exist within the literature examining the true impact of the ECHO model allowing for a more critical approach to interpreting and conducting future ECHO studies.

Alongside these strengths, this review also had its limitations. Firstly, this review was limited by its chosen time period. Due to 2020 being the cut-off point for studies, it excludes many newer studies that explored the ECHO model during the Covid-19 pandemic. As such studies were not included it cannot determine whether ECHO studies are employing some of the aforementioned outcome measures that were excluded in historical studies. This review also only included articles available in English and therefore may not be exhaustive.

## 6. Conclusions and future recommendations

ECHO has been successfully closing the knowledge gap between specialist and primary care, but it is undeniable that studies attempting to demonstrate the positive effects of ECHO contain their own gaps. Given the projected growth of digital healthcare it is expected that the ECHO model will continue to expand in its dissemination. While ECHO somewhat validates its improvements at improving confidence and knowledge with clinicians, there is a need for more patient centric-outcome measures to explore the offset effect ECHO has on specific, clinically relevant outcome measurements as well as in-depth cost comparators. Therefore, future recommendations include the need for more empirical and robust assessments of value within ECHO studies. The use of platforms like ECHO are more valued than ever before due to the consequences of the covid-19 pandemic. The pandemic highlighted the need for more digital and remote healthcare solutions. Henceforth, it is even more important that these solutions are appropriately assessed in terms of cost-effectiveness and that their impact to clinical outcomes are examined as they become more routine in chronic disease management.
